# Comprehensive evaluation of similarity between synthetic and real CT images for nasopharyngeal carcinoma

**DOI:** 10.1186/s13014-023-02349-7

**Published:** 2023-11-07

**Authors:** Siqi Yuan, Xinyuan Chen, Yuxiang Liu, Ji Zhu, Kuo Men, Jianrong Dai

**Affiliations:** National Cancer Center/National Clinical Research Center for Cancer/Cancer Hospital, Chinese Academy of Medical Sciences and Peking Union Medical College, Beijing, 100021 China

**Keywords:** Deep learning, Synthetic CT, Radiomics, Image similarity

## Abstract

**Background:**

Although magnetic resonance imaging (MRI)-to-computed tomography (CT) synthesis studies based on deep learning have significantly progressed, the similarity between synthetic CT (sCT) and real CT (rCT) has only been evaluated in image quality metrics (IQMs). To evaluate the similarity between synthetic CT (sCT) and real CT (rCT) comprehensively, we comprehensively evaluated IQMs and radiomic features for the first time.

**Methods:**

This study enrolled 127 patients with nasopharyngeal carcinoma who underwent CT and MRI scans. Supervised-learning (Unet) and unsupervised-learning (CycleGAN) methods were applied to build MRI-to-CT synthesis models. The regions of interest (ROIs) included nasopharynx gross tumor volume (GTVnx), brainstem, parotid glands, and temporal lobes. The peak signal-to-noise ratio (PSNR), mean absolute error (MAE), root mean square error (RMSE), and structural similarity (SSIM) were used to evaluate image quality. Additionally, 837 radiomic features were extracted for each ROI, and the correlation was evaluated using the concordance correlation coefficient (CCC).

**Results:**

The MAE, RMSE, SSIM, and PSNR of the body were 91.99, 187.12, 0.97, and 51.15 for Unet and 108.30, 211.63, 0.96, and 49.84 for CycleGAN. For the metrics, Unet was superior to CycleGAN (*P* < 0.05). For the radiomic features, the percentage of four levels (i.e., excellent, good, moderate, and poor, respectively) were as follows: GTVnx, 8.5%, 14.6%, 26.5%, and 50.4% for Unet and 12.3%, 25%, 38.4%, and 24.4% for CycleGAN; other ROIs, 5.44% ± 3.27%, 5.56% ± 2.92%, 21.38% ± 6.91%, and 67.58% ± 8.96% for Unet and 5.16% ± 1.69%, 3.5% ± 1.52%, 12.68% ± 7.51%, and 78.62% ± 8.57% for CycleGAN.

**Conclusions:**

Unet-sCT was superior to CycleGAN-sCT for the IQMs. However, neither exhibited absolute superiority in radiomic features, and both were far less similar to rCT. Therefore, further work is required to improve the radiomic similarity for MRI-to-CT synthesis.

*Trial registration*: This study was a retrospective study, so it was free from registration.

**Supplementary Information:**

The online version contains supplementary material available at 10.1186/s13014-023-02349-7.

## Background

Magnetic resonance imaging (MRI)-to-computed tomography (CT) image synthesis has been extensively researched because of its feasibility and potential [1–3]. The main purpose of MRI-to-CT synthesis is to replace CT with MRI acquisition. Synthetic CT is helpful for an MRI-only radiotherapy process, which offers the superior soft tissue contrast of MRI and makes up for the fact that MRI cannot be used for dose calculation [4–6]. The emerging MR-linear accelerator technology provides an application platform for synthetic CT.

Many studies have been conducted to demonstrate that synthetic CT based on deep learning can be effectively used in radiotherapy planning [7, 8] and image registration [9, 10]. Koike et al. employed synthetic CT with a mean absolute error (MAE) of 108.1 in treatment planning for brain radiotherapy. The differences in the dose relative to the prescribed dose were less than 1.0% [11]. Elizabeth et al. used a deep learning-derived synthetic CT instead of an MRI for MRI-CT, and CT-MRI deformable registration offered superior results to direct multimodal registration [12]. Many studies have attempted to reduce errors in synthetic images and improve their quality [13, 14]. Qi et al. simultaneously input images of T1, T2, T1-C, and T1 Dixon into a model, and the synthetic CT yielded a lower MAE than that of the single-channel MRI input [15]. Ladefoged et al. exploited the properties of UTE/ZTE and Dixon to provide a contrast of bone against air and fat against soft tissue, respectively, and obtained images with smaller errors than those obtained using only Dixon [16].

Although current MRI-to-CT studies have made significant progress, the evaluation of the similarity between sCT and rCT is limited to the low-dimensional information of images, such as grayscale and structure. Many studies have used subsequent image tasks to verify the quality of the synthetic images based on whether the generated images can replace the original images in the task or not [17, 18]. This indirect approach is not universal and standard, and it is prone to confusion. Unlike grayscale evaluation metrics, which are aimed at evaluating the image as a whole, radiomics pays more attention to local texture details, which are of greater significance in assessing the quality of the synthesized image in a more comprehensive assessment.

Certain studies have used the concordance correlation coefficient (CCC) to evaluate the similarity of radiomic features between sCT and rCT, demonstrating that deep learning methods can effectively improve the reproducibility of radiomic features between images [19–21]. This method can quantitatively reflect the degree of consistency between radiomic features. Such studies focused on the translation between the same modalities. For instance, Choe et al. [19] proposed a convolutional neural network (CNN) to reduce the difference between two chest CT images which were reconstructed from different kernels. Their results showed that the CNN could improve the reproducibility of radiomic features in pulmonary nodules or masses, which was beneficial for the generalizability of radiomics. Recent studies proved that multi-modality images were significant for radiomics. Combining the features of radiomic from different images, such as CT, MRI, and PET, would improve the prognostic performance for clinical application [22–25]. If cross-modal synthetic images that are consistent with the target images in terms of radiomics features can be obtained, it is expected to overcome problems such as the lack of cross-modal data in radiomics studies and promote the development of related research. This study was to perform a comprehensive evaluation in terms of image quality metrics (IQMs) and radiomic features for cross-modal synthetic images. A clear understanding of the similarity of image details can facilitate the improvement of synthetic images and wide clinical applications.

## Methods

### Data collection and preprocess

127 patients with nasopharyngeal carcinoma (NPC) from 2018 to 2021 were retrospectively analyzed in this study. Each of the patients underwent simulation CT scan (Philips Healthcare) without a contrast agent (parameters: voltage, 120 kV; exposure, 320 mAs; image size, 512 × 512 pixels; and slice thickness, 3 mm) and simulation MRI scan (3.0T MR, T1-FSE- Axial sequence, GE Healthcare) (parameters: repetition time, 834 ms; echo time, 7.96 ms; flip angle, 111°; image size, 512 × 512; and slice thickness, 3 mm) within the same day in the same position under the same pendulum fixation device. Each patient received a total dose of 70 Gy in 33 fractions. Target volume and organs at risk were contoured and verified independently by two radiation oncologists with over 8 years of experience treating NPC. The selected patients were required to undergo CT and MRI scans in the same position and there were no significant metal artifacts on CT images. Otherwise, the patient data would be excluded.

**Fig. 1:**
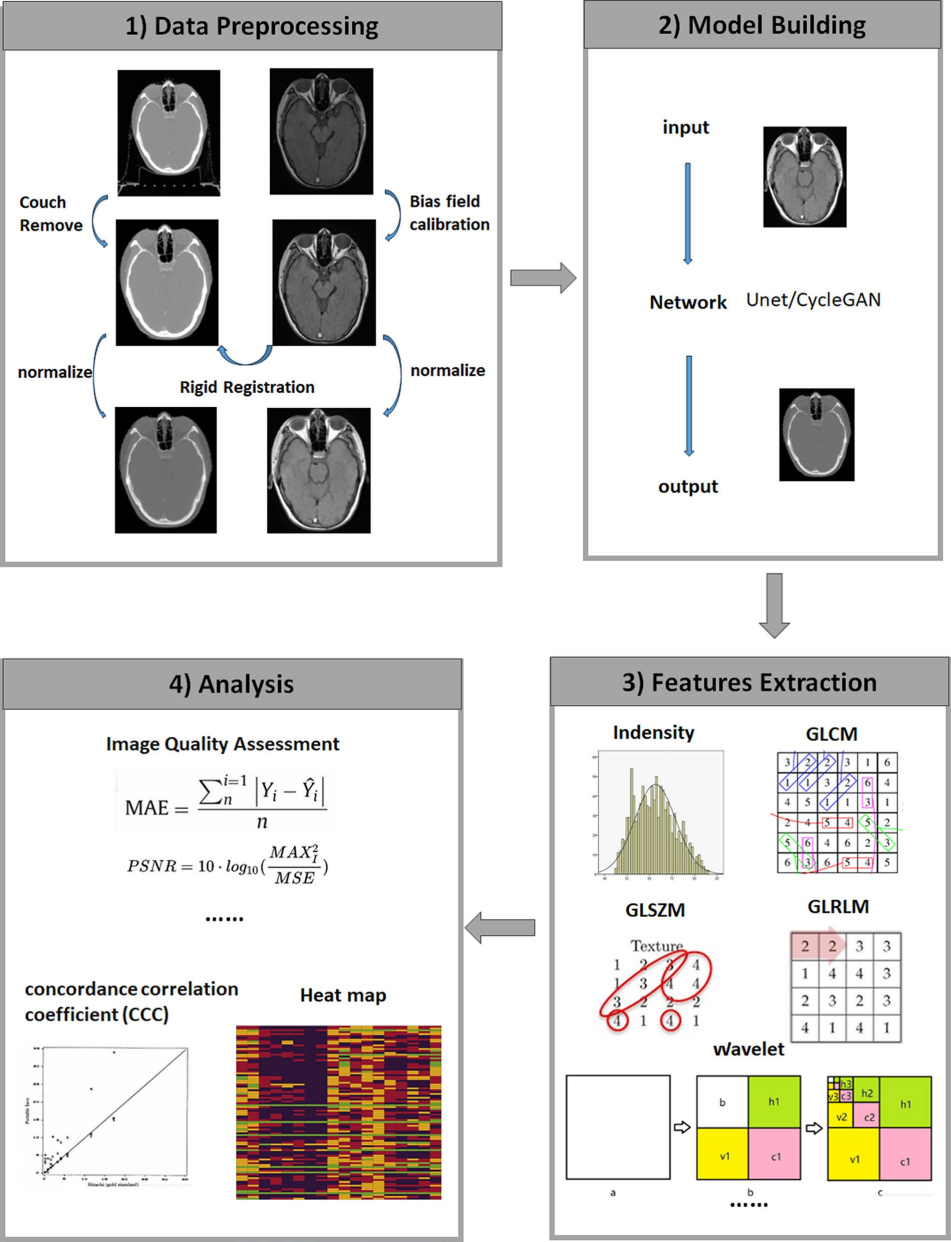
Schematic of the study. There were four main steps: (1) data processing; (2) model building; (3) feature extraction; and (4) analysis. For model building, we trained and tested two deep learning models (Unet and CycleGAN). Pyradiomics was applied for feature extraction

As shown in Fig. 1, the CT images need to remove the couch and the MRI need to calibrate the bias field for the data process. Rigid registration was performed on all pairs of CT and MRI by MIM software (Cleveland, OH, USA). Before feeding into the network, the images were normalized to [− 1, 1] by Min–Max normalization. The Institutional Review Board approval was obtained for this retrospective analysis, and the requirement to obtain informed consent was waived. All the patient data were deidentified.

### Deep learning methods

#### Unet

The Unet [26] used encoder–decoder architecture with long skip connections. Skip connections are added between each layer i and layer n − i, where n is the total number of layers. Each skip connection concatenates all channels at layer i with those at layer n − i. Down-sampling was implemented using 4 × 4 convolutional layers with a stride of 2, followed by a batch normalization layer and Leaky ReLU. In the encoder, there were eight such convolutional layers with filer numbers of [16,32,64,128,256,512,512,512] from input to bottleneck. For up-sampling, eight 4 × 4 transposed convolutional layers with a stride of 2, followed by a batch normalization layer and ReLU constituted the decoder. In the decoder, there were eight such convolutional layers with filer numbers of [512,512,256,128,64,32,16,1] from input to bottleneck. There was a Tanh activation function layer before output, and MSE was chosen as the loss function.

#### CycleGAN

For CycleGAN [27], two mappings were learned in the model coupled with two GANs: from MRI to CT and from CT to MRI. Chen et al. employed CycleGAN to generate synthetic kV-CT from megavoltage CT [28]. Based on the previously cited study, we chose the “CycleGAN-Resnet” for the present study, which contained nine residual blocks in the generator to minimize a residual, or error, image between two domains. A discriminator network adopted 70 × 70 PatchGAN, which was used to distinguish the true and false image blocks of 70 × 70 overlapping image blocks. When model training, The generator G_AB_ translates A to generate synthetic B as close as possible to real B, and the discriminator D_B_ distinguishes synthetic B from real B, which constitutes an adversarial loss. Then, synthetic B was translated to generate cycle A by G_BA_, where the cycle-consistent loss was employed to maintain the image structure of A.

### Experiments

The 127 patients were randomly divided into training (89 patients), validation (11 patients), and test sets (27 patients). These sets included 5853, 670, and 1534 pairs of two-dimensional (2D) images, respectively. For MRI-to-CT, the input and output were the 2D MR and CT images, respectively.

### Evaluation

#### Image quality

Four IQMs were used for evaluation, including peak signal-to-noise ratio (PSNR), MAE, root mean square error (RMSE), and structural similarity (SSIM). These metrics were also used for the evaluation of regions of interest (ROIs), including the GTVnx, brainstem, left parotid gland (Parotid L), right parotid gland (Parotid R), left temporal lobe (Temporal Lobe L), and right temporal lobe (Temporal Lobe R). Their definitions are available in Additional file 1: Appendix A.

#### Radiomic features

The radiation oncologists need to contour on the CT images after registration. Each patient was in the same position for CT and MRI scanning. For each patient, the contours of ROIs on the CT and MRI were same, and they were different between different patients. Therefore, the shape features were not included in the analysis. A total of 837 three-dimensional radiomic features including 18 first-order features, 75 texture features, and 744 (93*8) wavelet features were extracted from the ROIs with Pyradiomics [29] (an open-source program for radiomic analysis). A symmetrical matrix was used for the gray-level co-occurrence matrix. Other parameters in Pyradiomics were set to default values.

CCCs were used to evaluate the similarity of radiomic features between rCT and sCT. Following the classification in Chen et al. [30], and Lawrence et al. [31], the correlation degree of a feature was considered as excellent, good, moderate, or poor when CCC ≥ 0.9, 0.75 ≤ CCC < 0.9, 0.5 ≤ CCC < 0.75, and CCC < 0.5, respectively.

#### Statistical analysis

Data were analyzed using commercially available software, SPSS (IBM SPSS Statistics 25). The paired t-test was used to evaluate the significant difference in image quality and feature CCCs between Unet-sCT and CycleGAN-sCT. A p-value below 0.05 was considered to indicate a statistically significant difference.

## Results

### Image quality

**Fig. 2:**
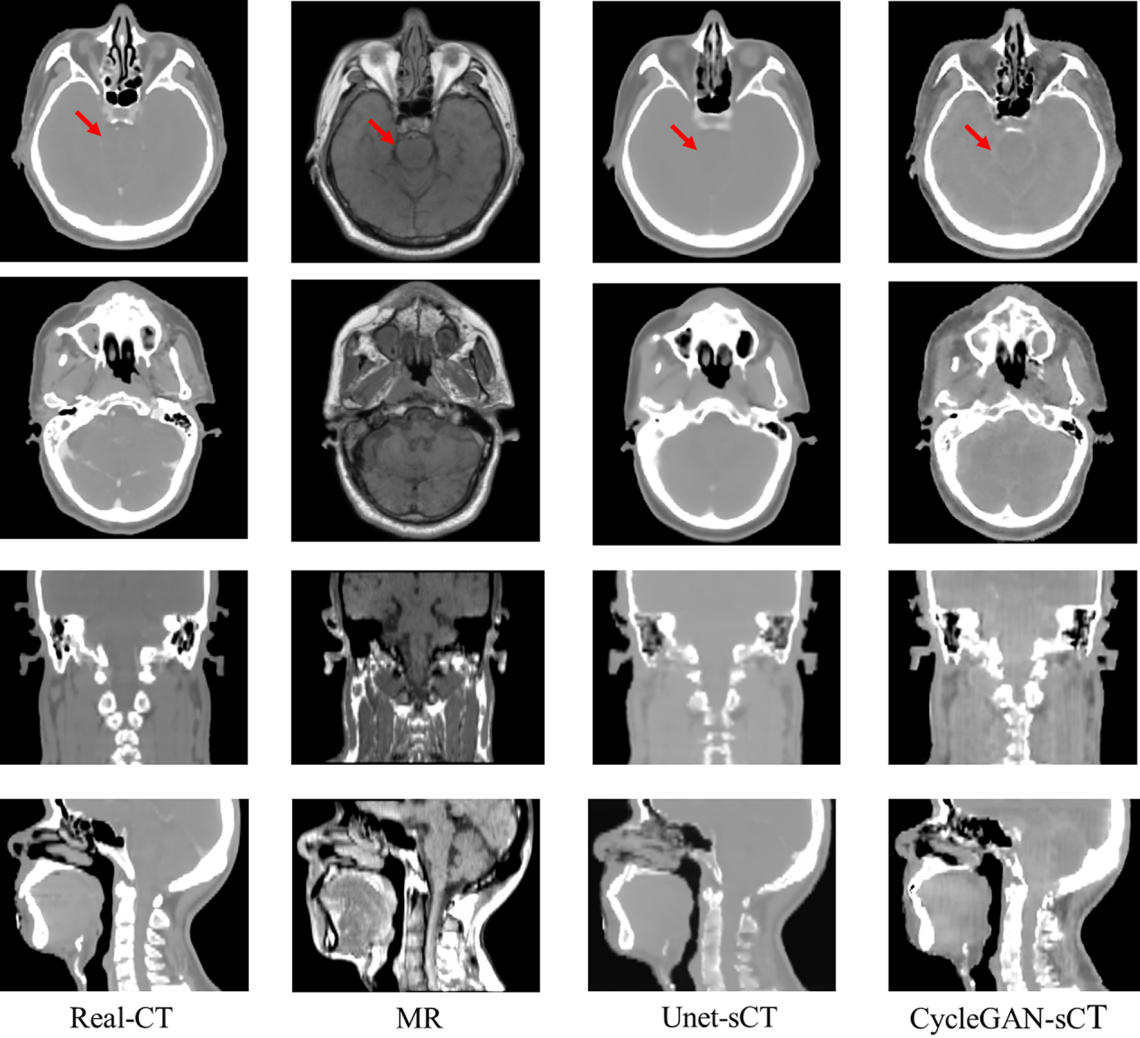
Real CT, MR, and synthetic images in axial, sagittal, and coronal positions. The window width and window level are 1000 and -60HU, respectively. The red arrow points out the location of the brain stem

Table 1 shows the evaluation results of the entire body and ROIs on MAE, RMSE, SSIM, and PSNR in Unet and CycleGAN, respectively. For the body, the MAE, RMSE, SSIM, and PSNR were 91.99, 187.12, 0.97, and 51.15 in Unet and 108.30, 211.63, 0.96, and 49.84 in CycleGAN. The former was superior to the latter in the aforementioned metrics in the body (*P* < 0.05). Figure 2 shows rCT, MR and synthesis CT of several layers. In both Unet and CycleGAN, complex bone structure information cannot be well learned. For soft tissue, such as the brain stem shown by the red arrow, GycleGAN retains boundary information similar to that in MRI, which could not be observed in Unet and real CT.

**Table 1:** Image quality evaluations of ROIs in Unet and CycleGAN

	Unet				CycleGAN			
	MAE(HU)	RMSE (HU)	SSIM	PSNR (dB)	MAE (HU)	RMSE (HU)	SSIM	PSNR (dB)
Body	91.99 ± 9.62^*^	187.12 ± 17.44^*^	0.97 ± 0.01^*^	51.15 ± 1.65^*^	108.30 ± 10.25	211.63 ± 19.98	0.96 ± 0.01	49.84 ± 2.11
GTVnx	114.69 ± 11.33^*^	175.47 ± 14.91^*^	0.93 ± 0.02^*^	52.66 ± 1.77^*^	149.31 ± 13.88	230.82 ± 21.1-	0.93 ± 0.01	50.33 ± 2.20
Brain Stem	6.14 ± 1.33^*^	9.32 ± 2.10^*^	0.96 ± 0.02^*^	77.92 ± 2.50^*^	46.819 ± 4.35	52.01 ± 5.76	0.92 ± 0.03	62.79 ± 2.66
Parotid L	25.42 ± 3.62^*^	40.86 ± 6.74^*^	0.88 ± 0.04^*^	65.29 ± 1.70^*^	37.74 ± 3.50	54.63 ± 5.93	0.90 ± 0.03	62.33 ± 2.56
Parotid R	28.94 ± 4.23^*^	43.08 ± 6.90^*^	0.83 ± 0.04^*^	62.51 ± 1.55^*^	47.93 ± 4.54	64.89 ± 6.15	0.78 ± 0.04	58.94 ± 2.50
TemporalLobe L	20.76 ± 2.10^*^	45.78 ± 7.44^*^	0.96 ± 0.01^*^	66.29 ± 1.65^*^	52.34 ± 5.06	81.93 ± 7.77	0.52 ± 0.07	59.00 ± 2.33
TemporalLobe R	16.20 ± 1.57^*^	35.22 ± 5.01^*^	0.98 ± 0.01^*^	66.62 ± 1.89^*^	39.43 ± 3.77	72.48 ± 6.88	0.76 ± 0.05	61.37 ± 2.42

*PSNR* peak signal-to-noise ratio, *MAE* mean absolute error, *RMSE* root mean square error, *SSIM* structural similarity index measurement, *Parotid L* left parotid gland, *Parotid R* right parotid gland, *Temporal Lobe L* left temporal lobe, *Temporal Lobe R* right temporal lobe

^*^Refer to *P* < 0.05 for paired t-test

To further understand the image similarity between rCT and sCT, we analyzed certain ROIs. Considering that GTVnx contains bone and air, the metrics of GTVnx were 114.69, 175.47, 0.93, and 52.66 in Unet and 149.31, 230.82, 0.93, and 50.33 in CycleGAN, which were worse than those of the entire body. The other ROIs were soft tissues, which exhibited better performance in most metrics. For the Temporal Lobe L, the SSIM in CycleGAN was only 0.52. A review of synthetic images shows that a portion of the Temporal Lobe L incorrectly learned a high-density structure, which could have affected the value of the SSIM.

### The CCCs of radiomic features between rCT and sCT

In GTVnx, the mean CCCs between Unet-sCT and rCT were 0.67 ± 0.21 for first-order, 0.73 ± 0.19 for texture, and 0.49 ± 0.25 for wavelet and 0.73 ± 0.21, 0.67 ± 0.22, and 0.63 ± 0.24 between CycleGAN-sCT and r-CT. There were no significant differences in the CCCs of all features between Unet and CycleGAN except wavelet (first-order *P* = 0.37, texture *P* = 0.55, and wavelet *P* < 0.05). For the other ROIs, the mean CCCs deteriorated to 0.43 ± 0.15, 0.354 ± 0.10, and 0.36 ± 0.08 in Unet and 0.188 ± 0.12, 0.192 ± 0.10, and 0.288 ± 0.09 in CycleGAN (Additional file 1: Table SA). The mean CCCs of the other ROIs in Unet were significantly larger than that of CycleGAN in the three category features (*P* < 0.05). The mean CCCs of the two models in the ROIs were satisfactory.

**Fig. 3:**
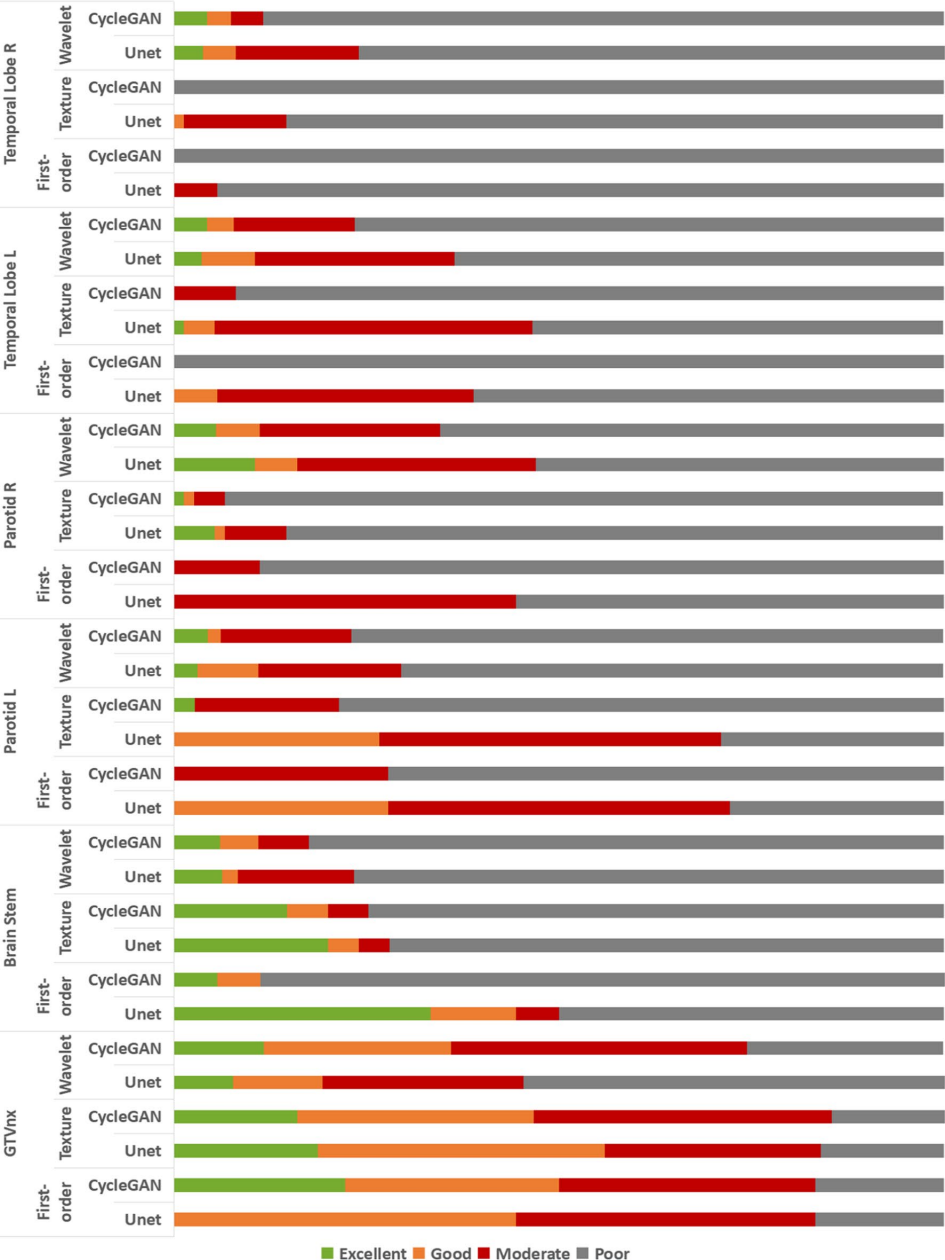
Distribution of CCC in different ROIs. Each ROI had three categories: first-order, texture, and wavelet. Each category had four levels: excellent, good, moderate, and poor for two models

Figure 3 shows the distribution of CCCs in different ROIs. For each ROI, the features were divided into three categories—first-order, texture, and wavelet. Each category exhibited four levels with different colors—green for excellent, yellow for good, red for moderate, and black for poor. Unet and CycleGAN were in adjacent bars with the same color and different patterns.

For GTVnx, 8.5%, 14.6%, 26.5%, and 50.4% of the total 837 features exhibited excellent, good, moderate, and poor correlations in Unet and 12.3%, 25%, 38.4%, and 24.4% in CycleGAN. For other ROIs, the value generally deteriorated to 5.44% ± 3.27%, 5.56% ± 2.92%, 21.38% ± 6.91%, and 67.58% ± 8.96% in Unet and 5.16% ± 1.69%, 3.5% ± 1.52%, 12.68% ± 7.51%, and 78.62% ± 8.57% in CycleGAN.

Overall, CycleGAN contained more features with excellent or good CCCs than Unet. In the original features (the features not belonged to the wavelet), a few features were poor, but more features were poor after the wavelet transformer. For the wavelet features, the CCC varied depending on the combination of high or low-frequency components. Wavelet features with high-frequency components tended to show relatively low CCCs. More details are shown as a heat map in Additional file 1: Fig. S1.

**Fig. 4:**
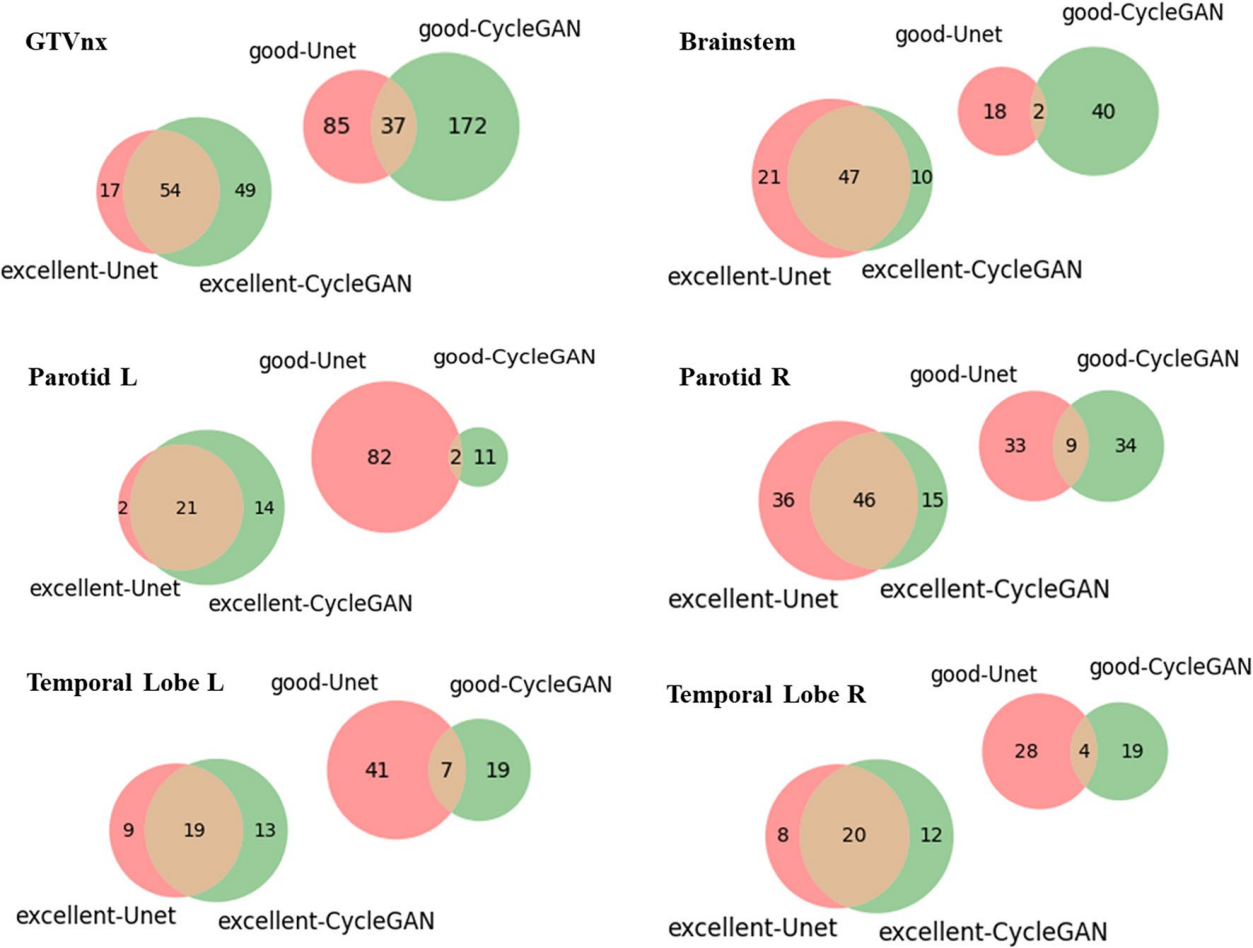
Venn diagrams illustrating overlaps in the excellent and good radiomic features for different ROIs in Unet and CycleGAN (red for Unet, green for CycleGAN)

The Venn diagrams in Fig. 4 illustrate the overlapped features in the two CCC classes (excellent and good) between the two cohorts of Unet (in red) and CycleGAN (in green) for different ROIs. In the excellent class, the proportion of overlapping features was more than 45% in all the ROIs. The ROIs were excellent in certain features, including GLRLM_GrayLevelNonUniformity, GLRLM_RunLengthNonUniformity, GLDM_DependenceNonUniformity, GLDM_GrayLevelNonUniformity, and NHTDM_Coarseness. However, in the good class, the proportion of overlapping features was less than 13% in all the ROIs, which indicated that the two models tended to learn different radiomic features. Additional details are listed in Additional file 1: Table SB.

In addition, 21 important features of GTVnx in the radiomic studies of NPC were collected through a literature search [32–37] (Additional file 1: Table SC), involving four tasks, including prognosis prediction, distant metastasis, local recurrence, and progression-free survival. For Unet, the 5/21(23.8%), 5/21(23.8%), 6/21(28.6%), and 5/21(23.8%) features were excellent, good, moderate, and poor, respectively, and 5/21(23.8%), 4/21(19.0%), 6/21(28.6%), and 6/21(28.6%) for CycleGAN. The percentage of excellent and good features in the 21 features was larger than that in all the features of GTVnx.

### Correlation between the MAE and mean CCC of the radiomic features

**Fig. 5:**
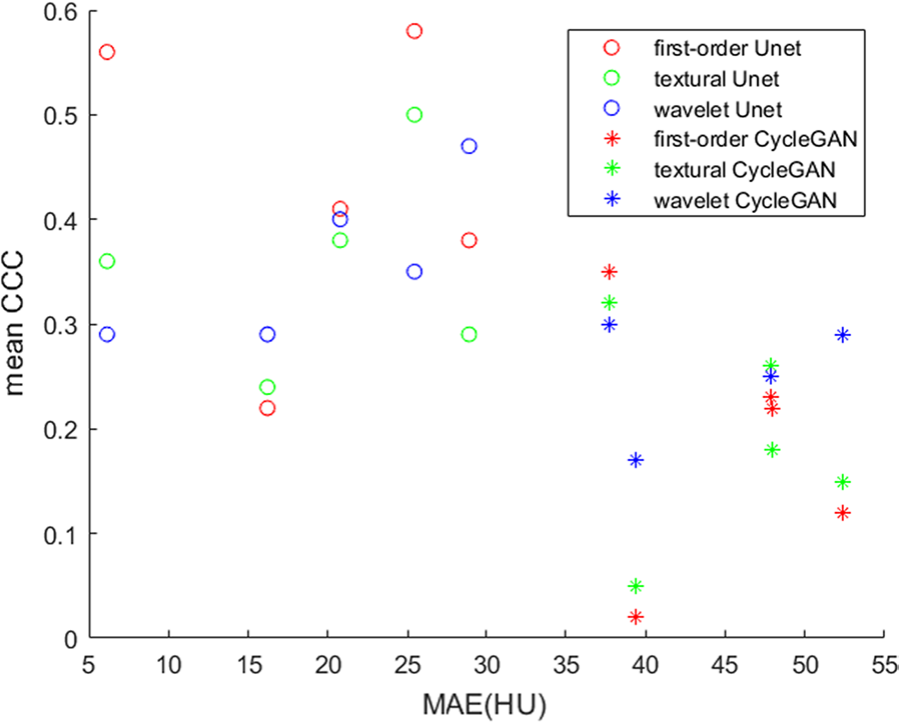
Scatter plot of MAE and the mean CCC. The x-axis represents MAE, and the y-axis represents the mean CCC of the three ROI categories. Each point indicates an ROI

Figure 5 shows the scatter plot of the MAE and mean CCC. For CycleGAN, the mean CCC of the first-order and texture features decreased with an increase in MAE. However, for Unet, the mean CCC of the wavelet exhibited a positive correlation with MAE. Anyway, in general, there was no strong regularity between the MAE and mean CCC, indicating that we could not demonstrate good radiomic-feature similarity for images with low MAE values. Thus, the quantitative evaluation of radiomic features on synthetic images is essential.

## Discussion

Here, we implemented the cross-modal image generation task of MRI-to-CT using two mainstream neural network models, Unet and CycleGAN. The image quality and radiomic features of the sCT were quantitatively evaluated. The results showed that only a small proportion of features exhibited excellent/good similarity. Therefore, current deep learning methods, whether supervised or unsupervised, could not effectively learn the radiomic features of target images in the cross-modal image synthesis task.

According to our knowledge, the MAE of the brain or head and neck was in the range of 67–131 HU with soft tissue less than 40 HU and bone/air exceeding 100 HU reported in several studies [11, 38–41]. Therefore, the MAE of the two models in this study were in the same order of magnitude as previous studies, which reflected the current average level of image synthesis. For MRI-to-CT synthesis, a main challenge is that the signal from bone tissue is weak for MRI, so the intensity of bone tissue on MRI is close to the air. For CT images, there is a positive correlation between the CT number and density. However, the density of bone tissue varies from patient to patient, which was difficult to reflect on the MRI. Besides, the GTV is highly heterogeneous, which contained air pockets (~ 8%), soft tissue (~ 63%) and bone tissue (~ 29%). The distributions of the CT number in GTV are various among different patients. So, the differences between the training set and testing set result in the high MAE. To get more accurate results in bone tissue and GTV, more data need to be collected to improve the performance, and we could try to introduce the clinic information or physical constraints to improve the performance.

Now many studies are concerned about the robustness of radiomic features and multi-modality studies [32–36]. Khadija Sheikh et al. [42] analyzed the CT/MR radiomic features to predict radiation-induced xerostomia after head-and-neck cancer radiotherapy. Compared with the result of AUC for CT only (0.69) and MR only (0.70), the multi-modality images CT and MR (0.75) improved a lot. Wenbing Lv et al. [36] also found that the prognostic performance of radiomics features from the PET/CT was better than the PET only or CT only for NPC patients. For head-and-neck cancer patients, there were many bone tissues around the tumor, while the MR images cannot provide much information. So, the CT images can provide additional information to help the radiomics modeling. Our study filled in the gaps of the reproducibility of synthetic CT generation.

In this study, we found that less than 40% of the features were excellent and good in GTVnx and 15% in other ROIs. In GTVnx, this deterioration in similarity in radiomic features was noticeable in the wavelet features compared to the original image features, which were sensitive to changes in image spatial and density resolutions. The results implied that current deep learning methods, supervised or unsupervised, could not effectively learn the radiomic features of target images in the cross-modal image synthesis task. Additional studies are required, such as improvements in network structure, to further improve the quality of synthetic images. We believe that this study provides a basis for image conversion using deep learning in radiomics and that it will help promote related research.

Our study has limitations. Although radiomic-feature similarity differences were measured, observed, and described in this study, certain underlying reasons were still not well understood or explained, particularly different similarities exhibited by different ROIs. Thus, further investigation is required. We simply stacked the 2D images to get 3D images. One common concern is the inter-slices differences after using the 2D network. We should improve it in the future.

Interestingly, the features learned by Unet and CycleGAN were considerably different, particularly the features that were “good” (over 88%). If the advantages of these two models could be combined, it would be possible to improve the radiomic feature similarity between synthetic and real images.

## Conclusions

Here, for the first time, to the best of our knowledge, we performed a comprehensive evaluation in terms of IQMs and radiomic features for sCT with two models, Unet and CycleGAN. Only a small fraction of features exhibited excellent and good similarity, highlighting the still unsolved problems of current image synthesis. The results of current MRI-to-CT image synthesis could not well contain the radiomic-feature information of the target image. Therefore, cross-modal image synthesis still requires further research and investigation to improve the similarity of radiomic features before it is applied to clinical radiomics.

### Supplementary Information

**Additional file 1. Appendix A:** Definition of indexes for image quality evaluation. **Supplementary Table A:** Mean CCCs of each region of interest except GTVnx. **Supplementary Table B:** The features exhibited excellent and good in both Unet  and CycleGAN for all regions of interest. **Supplementary Table C:** The 21 features of GTVnx in radiomics studies of NPC about four tasks including prognosis prediction, distant metastasis, local recurrence, and progression-free survival. **Fig S1:** CCC heat map of radiomic features in GTVnx.

## Data Availability

Research data are not available at this time.

## Abbreviations

sCT: Synthetic computerized tomography
rCT: Real computerized tomography
IQMs: Image quality metrics
ROI: Regions of interest
GTV: Gross tumor volume
PSNR: Peak signal-to-noise ratio
MAE: Mean absolute error
RMSE: Root mean square error
SSIM: Structural similarity index measurement
CCC: Concordance correlation coefficient
MRI: Magnetic resonance imaging
NPC: Nasopharyngeal carcinoma
Parotid L: Left parotid gland
Parotid R: Right parotid gland
Temporal Lobe L: Left temporal lobe
Temporal Lobe R: Right temporal lobe
