# Supplementary material for: Comprehensive evaluation of similarity between synthetic and real CT images for nasopharyngeal carcinoma
Source: Radiat Oncol. 2023 Nov 7;18:182. doi: 10.1186/s13014-023-02349-7 (PMC10629140; doi:10.1186/s13014-023-02349-7)
Supplement: Supplementary file 1 — Additional file 1. Appendix A: Definition of indexes for image quality evaluation. Supplementary Table A: Mean CCCs of each region of interest except GTVnx. Supplementary Table B: The features exhibited excellent and good in both Unet and CycleGAN for all regions of interest. Supplementary Table C: The 21 features of GTVnx in radiomics studies of NPC about four tasks including prognosis prediction, distant metastasis, local recurrence, and progression-free survival. Fig S1: CCC heat map of radiomic features in GTVnx. [file 13014_2023_2349_MOESM1_ESM.docx]

**Supplementary Material**

**Appendix A: Definition of indexes for image quality evaluation**

MAE is widely used and also is a simple full reference metric which is calculated by the absolute intensity differences of generated and reference image pixels. RMSE is similar to MAE in that it is a full-reference metric that measures the differences between the generated and reference image pixels. PSNR is used to calculate the ratio between the maximum possible signal power and the power of the distorting noise, where the reference image is regarded as the signal and the difference between the generated and reference image is regarded as the noise. Both MAE and PSNR are clear in physics for assessing the image quality. While SSIM is based on perception, where image degradation is regarded as the change of perception in structural information. It contains luminance masking, contrast masking and structure masking. These indexes include well-known quality metrics that measure the similarity between two images. Higher values of PSNR and SSIM imply good consistency between images. Conversely, values closer to zero are better for MAE and RMSE.

$PSNR=10\cdot{log}_{10} (\frac{{{MAX}_{I}}^{2}}{\frac{1}{N}\sum_{i=1}^{N} {(Y_{i}-X_{i})}^{2}})$ (C1)

$MAE =\frac{1}{N}\sum_{i=1}^{N} |Y_{i}-X_{i}|$ (C2)

$RMSE= \sqrt{\frac{\sum_{i=1}^{N} {(Y_{i}-X_{i})}^{2}}{N}}$ (C3)

$SSIM =\frac{(2m_{X}m_{Y}+0.01L) (2s_{XY}+0.03L)}{(m_{X}^{2}+m_{Y}^{2}+0.01L) (s_{X}^{2}+s_{Y}^{2}+0.03L)}$ (C4)

where *N* denotes the total number of voxels shared in common between the source *X* and the target *Y*. $X_{i}$ and $Y_{i}$ represent the intensity values of the $i$th voxel of the respective source. Additionally, $m_{x}$ and $s_{X}$ are the mean and variance of source X, $m_{Y}$ and $s_{Y}$ are the mirror case for another source Y, and $s_{xy}$ is the covariance of x and y. Additionally, 𝐿 is the range of the intensity. In this study, L is 4095. ${\mathrm{MAX}_{I}}^{2}$ represents the possible maximum pixel value of an image. For metrics of ROI calculation, the i refer to the volumes in ROI and N is the number of the volumes in ROI.

**Supplementary Table A**. Mean CCCs of each region of interest

|  | Unet | | | CycleGAN | | |
| --- | --- | --- | --- | --- | --- | --- |
|  | first-order | texture | wavelet | first-order | texture | wavelet |
| Brain Stem | 0.56 | 0.36 | 0.29 | 0.23 | 0.26 | 0.25 |
| Parotid L | 0.58 | 0.50 | 0.35 | 0.35 | 0.32 | 0.30 |
| Parotid R | 0.38 | 0.29 | 0.47 | 0.22 | 0.18 | 0.43 |
| Temporal Lobe L | 0.41 | 0.38 | 0.40 | 0.12 | 0.15 | 0.29 |
| Temporal Lobe R | 0.22 | 0.24 | 0.29 | 0.02 | 0.05 | 0.17 |

**Supplementary Table B**. The features exhibited excellent and good in both Unet and CycleGAN for all regions of interest

| **Excellent** |  | Ori* | w-*LLH | w-LHL | w-LHH | w-HLL | w-HLH | w-HHL | w-HHH | w-LLL |
| --- | --- | --- | --- | --- | --- | --- | --- | --- | --- | --- |
| First-  order | Energy |  | G* |  |  |  |  |  |  |  |
|  | Minimum | BS* |  |  |  |  |  |  |  | BS |
| GLCM | Autocorrelation | BS |  |  |  |  |  |  |  | BS |
|  | ClusterProminence | BS |  |  |  |  |  |  |  | BS |
|  | ClusterShade | BS |  |  |  |  |  |  |  | BS |
|  | JointAverage | BS |  |  |  |  |  |  |  | BS |
|  | SumAverage | BS |  |  |  |  |  |  |  | BS |
| GLRLM | GrayLevelNonUniformity | G | G/PR* | G/BS/PL*/PR | G/BS/  PL/PR/TL/TR | G/BSPL/PR/TL* | G/BS/PL/PR/TL/TR* | G/BS/PL/PR/TL/TR | G/BS/PL/PR/TL/TR | G |
|  | HighGrayLevelRunEmphasis | BS |  |  |  |  |  |  |  | BS |
|  | LongRunLowGrayLevelEmphasis |  |  |  |  |  |  |  | PR |  |
|  | LowGrayLevelRunEmphasis |  | PR |  |  |  |  |  |  |  |
|  | RunLengthNonUniformity |  | G | G | G/PR/  TR | G | GPR/TL/TR | G/BS/TL/TR | G/BS/PL/PR/TL/TR | G |
|  | ShortRunHighGrayLevelEmphasis |  |  |  |  |  |  |  |  | BS |
|  | ShortRunLowGrayLevelEmphasis |  | PR |  |  |  |  |  |  |  |
| GLSZM | GrayLevelNonUniformity | G | G | G | G | G | G | G | G | G |
|  | GrayLevelNonUniformityNormalized |  | PR |  |  |  |  |  |  |  |
|  | HighGrayLevelZoneEmphasis | BS |  |  |  |  |  |  |  | BS |
|  | LargeAreaEmphasis |  |  |  |  |  |  |  | PR |  |
|  | LargeAreaLowGrayLevelEmphasis |  |  |  |  |  |  |  | PR |  |
|  | LowGrayLevelZoneEmphasis |  | PR |  |  |  | PR |  | PR |  |
|  | SizeZoneNonUniformity | G | G |  |  |  |  |  |  | G |
|  | SmallAreaHighGrayLevelEmphasis |  |  |  |  |  |  |  |  | BS |
|  | SmallAreaLowGrayLevelEmphasis |  | PR |  |  |  | PR |  |  |  |
|  | ZoneVariance |  |  |  |  |  |  |  | PR |  |
| GLDM | DependenceEntropy |  |  |  |  |  |  |  |  | G |
|  | DependenceNonUniformity | G | G | PL/PR | G/PR | G/PL/PR | G/PR/TR | G/BS/PR/TL/TR | G/BS/PR/TL/TR | G |
|  | GrayLevelNonUniformity |  | G/BS/PL | BS/PL | BS/PL/  PR/TL/TR | G/BS | G/BS/PR/TL/TR | G/BS/PL/PR/TL/TR | G/BS/PL/PR/TL/TR |  |
|  | HighGrayLevelEmphasis | BS |  |  |  |  |  |  |  | BS |
|  | LargeDependenceHighGrayLevelEmphasis | BS |  |  |  |  |  |  |  |  |
|  | LargeDependenceLowGrayLevelEmphasis |  | PR |  |  |  |  |  | PR |  |
|  | LowGrayLevelEmphasis |  | PR |  |  |  |  |  | PR |  |
| NHTDM | Busyness |  | PR |  |  |  |  |  | BS/PR |  |
|  | Coarseness | G/PR | G/PR | G/PR/PL | G/BS/PL/PR/TL/TR | G/BS/PL/PR/TL/TR | G/BS/PL/PR/TL/TR | G/BS/PL/PR/TL/TR | G/BS/PL/PR/TL/TR | G |
|  | Complexity |  |  |  |  |  |  |  |  |  |
|  | Contrast | BS |  |  |  |  |  |  |  | BS |
|  | Strength |  |  |  |  |  |  |  | BS |  |

*ori was original

*w-* was wavelet-*.

*G was GTVnx, BS was Brainstem, PR was Parotid R, PL was Parotid L, TR was Temporal Lobe R, TL was Temporal Lobe L.

**Supplementary Table B (Continuation table):** The features exhibited excellent and good in both Unet and CycleGAN for all regions of interest

| **Good** |  | Ori* | w*t-LLH | w-LHL | w-LHH | w-HLL | w-HLH | w-HHL | w-HHH | w-LLL |
| --- | --- | --- | --- | --- | --- | --- | --- | --- | --- | --- |
| First-order | Entropy |  | G |  |  |  |  |  |  | G |
|  | InterquartileRange | G |  |  |  |  |  |  |  | G |
|  | Mean | G |  |  |  | G |  |  |  | G |
|  | RobustMeanAbsoluteDeviation | G |  |  |  |  |  |  |  | G |
|  | Uniformity |  | G |  |  |  |  |  |  |  |
| GLCM | Correlation |  |  |  |  |  |  |  |  | G |
|  | DifferenceAverage |  |  |  |  |  | TR |  |  |  |
|  | DifferenceEntropy | G | G |  |  |  |  |  |  |  |
|  | Id |  |  |  |  |  | TL/TR |  |  |  |
|  | Idm |  |  |  |  |  | TL/TR |  |  |  |
|  | Idn | G |  |  |  |  |  |  |  |  |
|  | Imc1 |  |  |  |  | G | PR | PL |  | G |
|  | Imc2 |  |  |  |  |  | PR |  | PR |  |
|  | JointEntropy |  | G |  |  |  |  |  |  | G |
|  | MCC |  |  |  |  |  | PR |  |  |  |
|  | SumEntropy | G |  |  |  |  |  |  |  | G |
| GLRLM | GrayLevelNonUniformityNormalized | G | G |  |  |  |  |  |  |  |
|  | HighGrayLevelRunEmphasis | G |  |  |  |  |  |  |  |  |
|  | RunLengthNonUniformity |  |  | TL |  |  |  | PL |  |  |
|  | RunLengthNonUniformityNormalized |  |  |  |  |  | TL |  |  |  |
|  | RunPercentage |  |  |  |  |  | TL |  |  |  |
|  | ShortRunHighGrayLevelEmphasis | G |  |  |  |  |  |  |  |  |
| GLSZM | GrayLevelNonUniformity |  |  |  |  | TL |  |  |  |  |
|  | GrayLevelNonUniformityNormalized |  |  |  |  | PR |  |  |  |  |
|  | LargeAreaHighGrayLevelEmphasis |  |  |  | G | G |  | G | G |  |
|  | ZoneEntropy | G | PR |  | PR |  |  | PR |  | G |
|  | ZonePercentage | G | G |  |  |  |  |  |  | G |
| GLDM | DependenceEntropy |  | G | G |  |  |  |  |  |  |
|  | DependenceNonUniformity |  |  | TL/TR |  |  | BS |  |  |  |
|  | DependenceNonUniformityNormalized |  |  |  |  |  |  |  |  | G |
|  | GrayLevelNonUniformity |  |  | PR |  |  |  |  |  |  |
|  | SmallDependenceEmphasis |  | G |  | BS |  |  |  |  |  |

*ori was original

*w-* was wavelet-*.

*G was GTVnx, BS was Brainstem, PR was Parotid R, PL was Parotid L, TR was Temporal Lobe R, TL was Temporal Lobe L.

**Supplementary Table C**. The 21 features of GTVnx in radiomics studies of NPC about four tasks including prognosis prediction, distant metastasis, local recurrence, and progression-free survival.

|  |  | Unet | CycleGAN |
| --- | --- | --- | --- |
| Prognostic | original_firstorder_Energy | Good | Excellent |
|  | original_glcm_MaximumProbability | Good | Poor |
|  | original_glrlm_GrayLevelNonUniformity | Excellent | Excellent |
|  | original_glrlm_ShortRunLowGrayLevelEmphasis | Poor | Poor |
|  | original_glszm_GrayLevelNonUniformity | Excellent | Excellent |
|  | wavelet-LLH_glcm_DifferenceVariance | Moderate | Poor |
|  | wavelet-LLH_ngtdm_Complexity | Moderate | Poor |
|  | wavelet-LHL_glcm_Idmn | Moderate | Good |
|  | wavelet-LHL_glrlm_LongRunLowGrayLevelEmphasis | Poor | Moderate |
|  | wavelet-LHL_glrlm_ShortRunHighGrayLevelEmphasis | Poor | Moderate |
|  | wavelet-LHH_glszm_GrayLevelVariance | Poor | Moderate |
|  | wavelet-HLH_glrlm_GrayLevelNonUniformity | Excellent | Excellent |
|  | wavelet-HHH_glszm_ZonePercentage | Poor | Moderate |
|  | wavelet-HHH_ngtdm_Complexity | Moderate | Poor |
|  | wavelet-LLL_glcm_SumEntropy | Good | Good |
| Distant metastases | original_glrlm_LongRunHighGrayLevelEmphasis | Good | Poor |
|  | original_glszm_SizeZoneNonUniformity | Excellent | Excellent |
|  | original_glszm_ZoneVariance | Good | Moderate |
| Local recurrence | original_glcm_Correlation | Moderate | Good |
|  | original_glszm_LowGrayLevelZoneEmphasis | Moderate | Moderate |
| Progress free sverial | original_ngtdm_Strength | Excellent | Good |


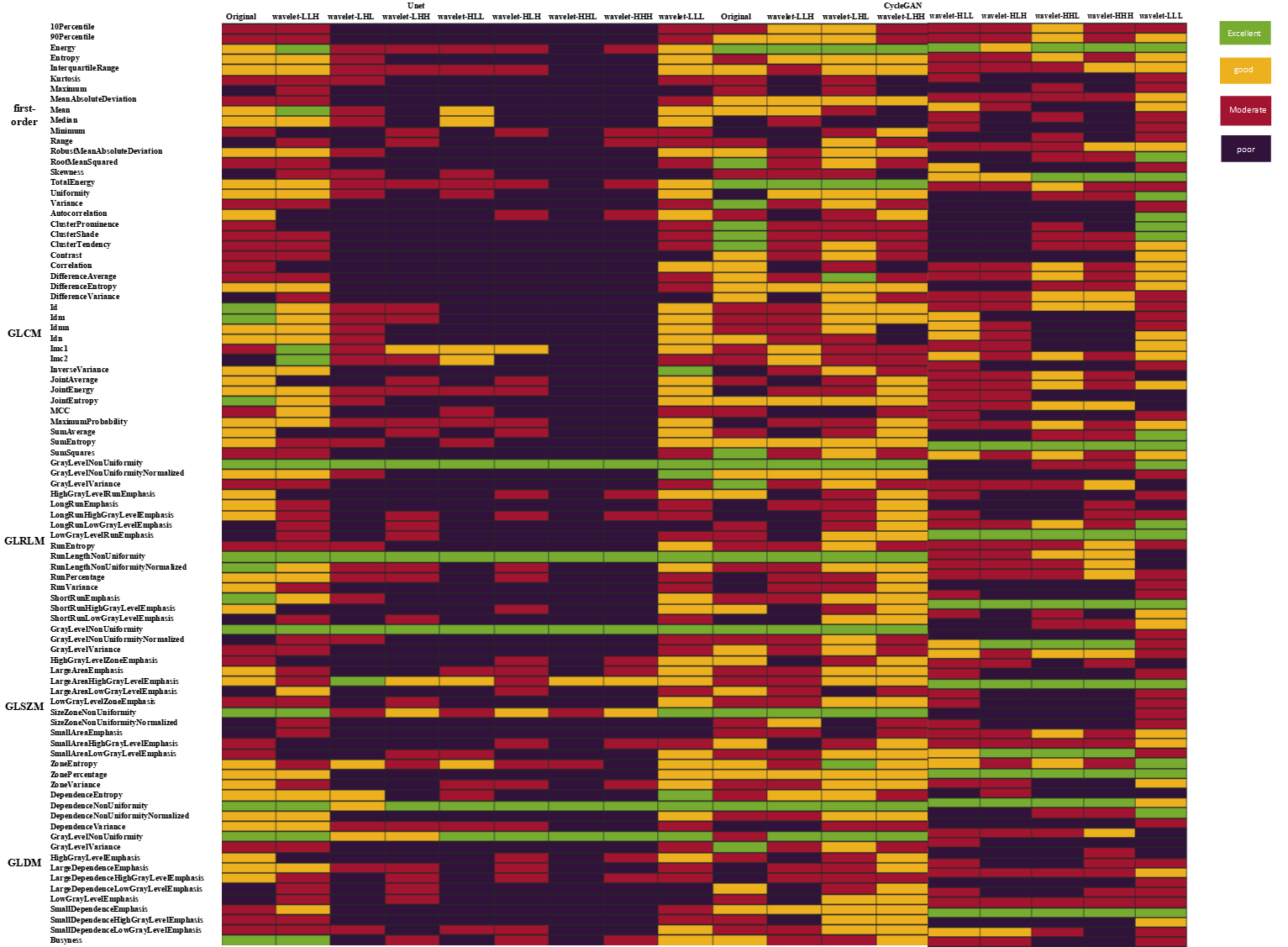


**Figure S1.** CCC heat map of radiomic features in GTVnx. The heat map of CCCs for original features and images after eight wavelet transformers between Unet-sCT/CycleGAN-sCT and rCT. CycleGAN exhibited better radiomic-feature similarity.
